# Pre-pregnancy BMI-associated miRNA and mRNA expression signatures in the placenta highlight a sexually-dimorphic response to maternal underweight status

**DOI:** 10.1038/s41598-021-95051-1

**Published:** 2021-08-03

**Authors:** Jeliyah Clark, Lauren A. Eaves, Adriana R. Gaona, Hudson P. Santos, Lisa Smeester, Jacqueline T. Bangma, Julia E. Rager, T. Michael O’Shea, Rebecca C. Fry

**Affiliations:** 1 Department of Environmental Sciences and Engineering, Gillings School of Global Public Health, The University of North Carolina at Chapel Hill, Chapel Hill, NC USA; 2 The Institute for Environmental Health Solutions, Gillings School of Global Public Health, The University of North Carolina at Chapel Hill, Chapel Hill, NC USA; 3 School of Nursing, University of North Carolina, Chapel Hill, NC USA; 4 Curriculum in Toxicology and Environmental Medicine, School of Medicine, University of North Carolina, Chapel Hill, NC USA; 5 Division of Neonatal-Perinatal Medicine, Department of Pediatrics, University of North Carolina, Chapel Hill, NC USA

**Keywords:** Developmental biology, Genetics, Systems biology

## Abstract

Pre-pregnancy body mass index (BMI) is associated with adverse pregnancy and neonatal health outcomes, with differences in risk observed between sexes. Given that the placenta is a sexually dimorphic organ and critical regulator of development, examining differences in placental mRNA and miRNA expression in relation to pre-pregnancy BMI may provide insight into responses to maternal BMI in utero. Here, genome-wide mRNA and miRNA expression levels were assessed in the placentas of infants born extremely preterm. Differences in expression were evaluated according to pre-pregnancy BMI status (1) overall and (2) in male and female placentas separately. Overall, 719 mRNAs were differentially expressed in relation to underweight status. Unexpectedly, no genes were differentially expressed in relation to overweight or obese status. In male placentas, 572 mRNAs were associated with underweight status, with 503 (70%) overlapping genes identified overall. Notably, 43/572 (8%) of the mRNAs associated with underweight status in male placentas were also gene targets of two miRNAs (*miR-4057* and *miR-128-1-5p*) associated with underweight status in male placentas. Pathways regulating placental nutrient metabolism and angiogenesis were among those enriched in mRNAs associated with underweight status in males. This study is among the first to highlight a sexually dimorphic response to low pre-pregnancy BMI in the placenta.

## Introduction

Pre-pregnancy weight status is a risk factor for adverse maternal and perinatal outcomes. Overweight and obese pregnant women are at an increased risk of intrauterine fetal demise, fetal macrosomia, and delivery of an infant admitted to the neonatal intensive care unit^1–3^. This population also experiences an increased risk of developing gestational diabetes, pre-eclampsia, and other pregnancy-induced cardiometabolic disorders^1^. Pregnancy and neonatal complications associated with pre-pregnancy underweight status are less frequently highlighted. Nevertheless, being underweight during pregnancy is also a risk factor for perinatal outcomes such as intrauterine growth restriction (IUGR), low birth weight, and preterm delivery^1,2,4^. As the primary interface between mother and fetus, the placenta represents an ideal target organ for investigating molecular mechanisms underpinning these associations.

The placenta is the first complex organ to form during pregnancy and serves many critical functions, including providing oxygen and nutrients to the developing fetus while removing waste^5–7^. The placenta also contributes to the regulation of hormones impacting parturition, metabolism, and fetal growth throughout the progression of pregnancy^5^. An increasing body of evidence supports that male and female placentas respond to and interact with nutritional and other environmental stimuli differently, setting the stage for sex-specific patterns of health outcomes at birth and later-in-life. Indeed, the placenta displays sexually dimorphic patterns of protein expression, gene and miRNA expression, CpG methylation and other physiologic characteristics^8–12^. Sex-specific changes in epigenetic marks also occur post-fertilization in response to environmental conditions. As an example, differential expression of placental miRNAs implicated in adipogenesis has been observed in placentas collected from females but not males^13^. Given that placentas derived from males and females display differences in their responses to a variety of adverse in utero environments and prenatal stressors^11,14–17^, it is important to study whether the relationship between pre-pregnancy BMI and placental gene expression varies between placentas derived from male and female births.

Given their relative plasticity, transcriptomic and epigenomic signatures provide a valuable tool for assessing the role of the placenta in mediating the relationship between pre-pregnancy BMI and perinatal outcomes. Candidate gene association studies have previously identified placental miRNAs associated with pre-pregnancy BMI^13,18,19^. However, to date no study has integrated genome-wide placental mRNA and miRNA expression profiles in the investigation of mechanisms underlying pre-pregnancy BMI-associated health outcomes. Using one of the largest existing placental -omics datasets, we performed differential gene expression analyses to characterize differences in mRNA and miRNA expression according to pre-pregnancy BMI status in the Extremely Low Gestational Age Newborns (ELGAN) study. These data were analyzed in relation to all subjects, as well as among male and female placentas separately. We hypothesized that differential mRNA and miRNA expression profiles would be identified in placentas derived from underweight, overweight, and obese women, as compared to normal weight women. Additionally, we hypothesized that unique BMI-associated mRNA and miRNA expression signatures would be identified in placentas collected from male and female infants.

## Results

### Participant characteristics

Participant information is summarized for the subjects from whom the 390 placentas are available for use in the current study (Table 1). The current study is composed of placentas collected from women aged 14–45 years old. Most women self-identified as White (61.4%), had a normal BMI ranging from 18.5 to 25.0 kg/m^2^ (51.8%), were not exposed to first- or secondhand smoking (76.4%), and completed between 12 and 16 years of education (48.8%). Placentas were collected from 205 (52.6%) male infants, 185 (47.4%) female infants, and the average pregnancy ended at 26 weeks. After exclusions for covariate missingness, potential outliers, and low RNA expression, 360 placentas were available for mRNA and miRNA analyses (Table 1). Similar demographic distributions were apparent in the final sets of subjects included in the mRNA and miRNA analyses as compared to the 390 available for analysis.

**Table 1:** Distributions of select characteristics among study participants, Extremely Low Gestational Age Newborns Cohort, 2002–2004. Maternal demographic data, pregnancy characteristics, and data on birth outcomes are presented for the ELGAN subjects used in each analysis. Data are presented as the number (%) of subjects and mean [range] in the cohort.

	Overall (N = 390)	miRNA subset (N = 360)	mRNA subset (N = 360)
Maternal age (years)	29 [14–45]	29 [14–45]	29 [14–45]
**Maternal race**			
White	237 (61.4)	221 (61.4)	222 (61.7)
Non-White	149 (38.6)	139 (38.6)	138 (38.3)
**Maternal BMI (kg/m**^**2**^**)**			
Underweight (< 18.5)	34 (8.3)	26 (7.2)	26 (7.2)
Normal (18.5 to < 25.0)	213 (51.8)	193 (53.6)	195 (54.2)
Overweight (25.0 to < 30.0)	77 (18.7)	67 (18.6)	66 (18.3)
Obese (> 30.0)	87 (21.2)	74 (20.6)	73 (20.3)
Smoke exposure*			
No	292 (76.4)	276 (76.7)	276 (76.7)
Yes	90 (23.6)	84 (23.3)	84 (23.3)
**Gestational weight gain (lbs)**	22.8 [− 23.0, 80.0]	22.9 [− 23.0, 74.0]	22.9 [− 23.0, 74.0]
**Highest level of educational attainment**			
Less than 12 years	49 (12.9)	45 (12.5)	44 (12.2)
Between 12 and 16 years	185 (48.8)	174 (48.3)	175 (48.6)
Greater than 16 years	145 (38.3)	141 (39.2)	141 (39.2)
**Newborn sex**			
Male	205 (52.6)	190 (52.8)	190 (52.8)
Female	185 (47.4)	170 (47.2)	170 (47.2)
**Gestational age (weeks)**	26 [23–27]	25 [23–27]	25 [23–27]

*First- or second-hand smoke exposure.

### Pre-pregnancy underweight status is associated with placental mRNA expression in male placentas

Placental mRNA expression levels were evaluated in relation to pre-pregnancy BMI. Specifically, placental mRNA expression profiles of underweight (N = 26), overweight (N = 66), and obese (N = 73) women were compared to placental mRNA expression profiles of normal weight women (N = 195). No genes were significantly (FDR p-value < 0.10) differentially expressed in relation to overweight or obese status. In contrast, 719 mRNAs were identified with expression levels significantly associated with underweight status overall (e.g., non-sex stratified analysis) (Table S1).

**Figure 1:**
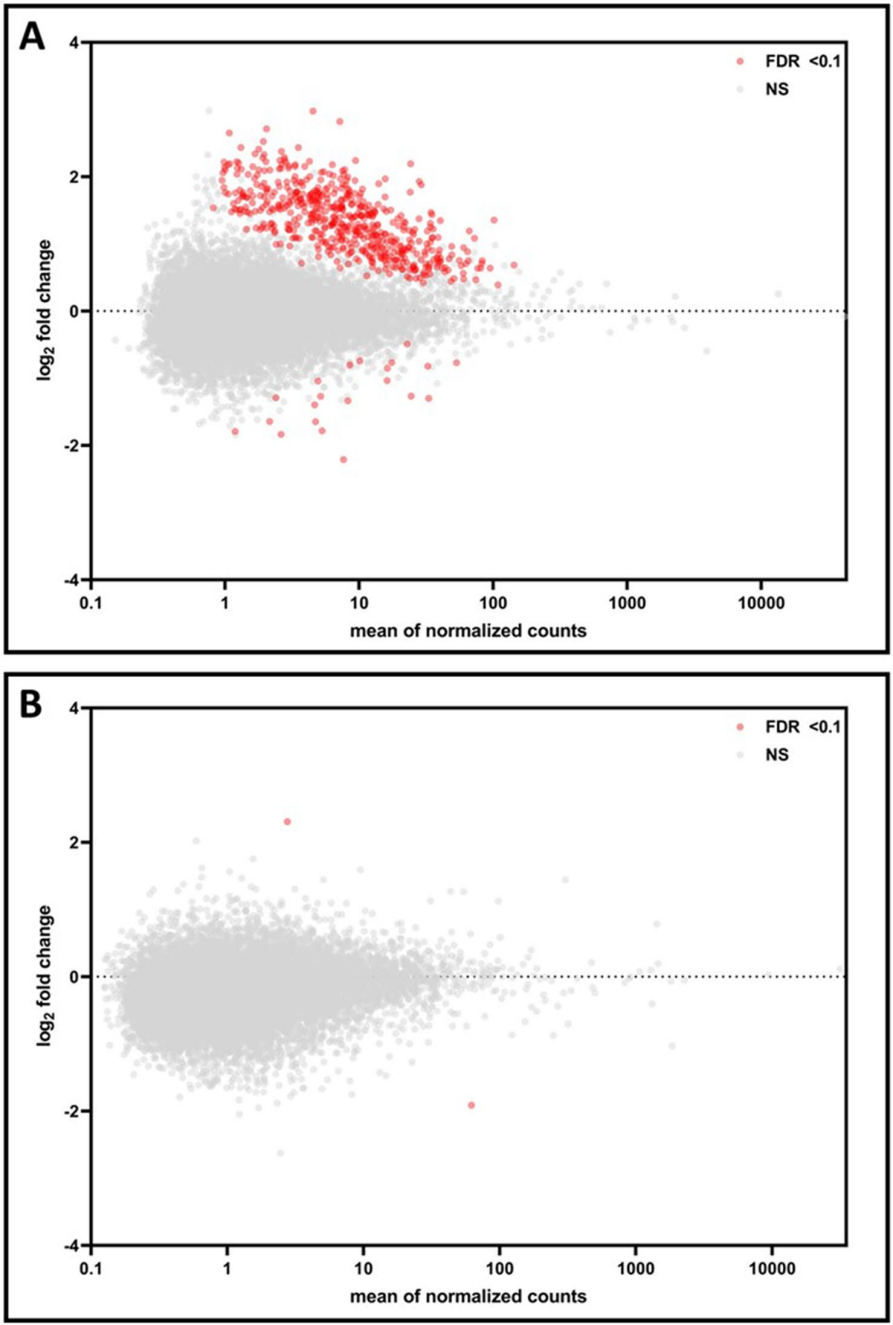
Log_2_-fold changes in mRNA expression associated with underweight status vs. mean of normalized counts. log_2_-fold changes represent the change in mRNA expression in (**A**) male and (**B**) female placentas. Data points colored in red indicate genes significantly (FDR p-value < 0.1) associated with underweight status. Genes not significantly associated with underweight status are displayed in grey.

In evaluating placentas collected from male and female births separately, evidence for sexual dimorphism was observed. Specifically, in placentas collected from male births, 572 mRNAs were associated with underweight status, 503 of these genes overlapped with the 719 identified overall (Fig. 1A, Table S2). Most (96%) of the genes identified in male placentas displayed higher expression levels (log_2_-fold change (FC) > 0) in relation to underweight status. In placentas collected from female births, 2 mRNAs were associated with underweight status (Fig. 1B, Table S2). These included Cytochrome P450 Family 1 Subfamily B Member 1 (*CYP1B1*) (log_2_-FC = 2.31, FDR p-value = 0.08) and microRNA 1248 (*MIR1248*) (log_2_-FC = − 1.91, FDR p-value = 0.08). Genes differentially expressed in male placentas were also expressed in female placentas (Table S3), but no association with underweight status was observed.

### Pathways, diseases, and functions enriched for mRNAs associated with underweight status

Pathway and network analyses were performed to identify canonical pathways, diseases, and functions enriched for genes found to be differentially expressed in relation to underweight status in male placentas. Genes displaying higher (log_2_-FC > 0) and lower (log_2_-FC < 0) expression in relation to underweight status were evaluated separately. Genes displaying higher expression in relation to underweight status overlapped with known gene regulatory networks (Table S4). These included: EIF2 Signaling (p-value = 4.31E−09, Overlap: 23/224), Regulation of eIF4 and p70S6K Signaling (p-value = 9.81E−05, Overlap: 13/157), ATM Signaling (p-value = 1.02E−04, Overlap: 10/97), Prolactin Signaling (p-value = 1.27E−04, Overlap: 9/81), Protein Kinase A Signaling (p-value = 2.41E−04, Overlap: 22/399), Insulin Receptor Signaling (p-value = 5.19E−04, 11/140), VEGF Signaling (p-value = 5.78E−04, Overlap: 9/99), VEGF Family Ligand–Receptor Interactions (p-value = 8.51E−04, Overlap: 8/84), IGF-1 Signaling (p-value = 3.34E−03, Overlap: 8/104), and mTOR Signaling (p-value = 1.58E−03, Overlap: 13/210). Several of these pathways were conserved when filtering for findings or molecules where cell/tissue/organ was specified as the placenta. These included: Angiopoietin Signaling (p-value = 1.32E−02, Overlap: 6/42), Thrombin Signaling (p-value = 1.85E−02, Overlap: 11/114), Insulin Receptor Signaling (p-value = 3.66E−02, Overlap: 8/81), and EIF2 Signaling (p-value = 4.04E−02, Overlap: 10/113).

Pathways enriched among genes displaying lower expression in relation to underweight status among males included: Wnt/b-catenin Signaling (p-value = 8.13E−03, Overlap: 2/173), RhoGDI Signaling (p-value = 8.77E−03, Overlap: 2/180), and Caveolar-mediated Endocytosis Signaling (p-value = 1.50E−03, Overlap 2/73) (Table S4). Some of these pathways were also conserved when filtering for findings or molecules where the cell/tissue/organ was specified as the placenta.

**Figure 2:**
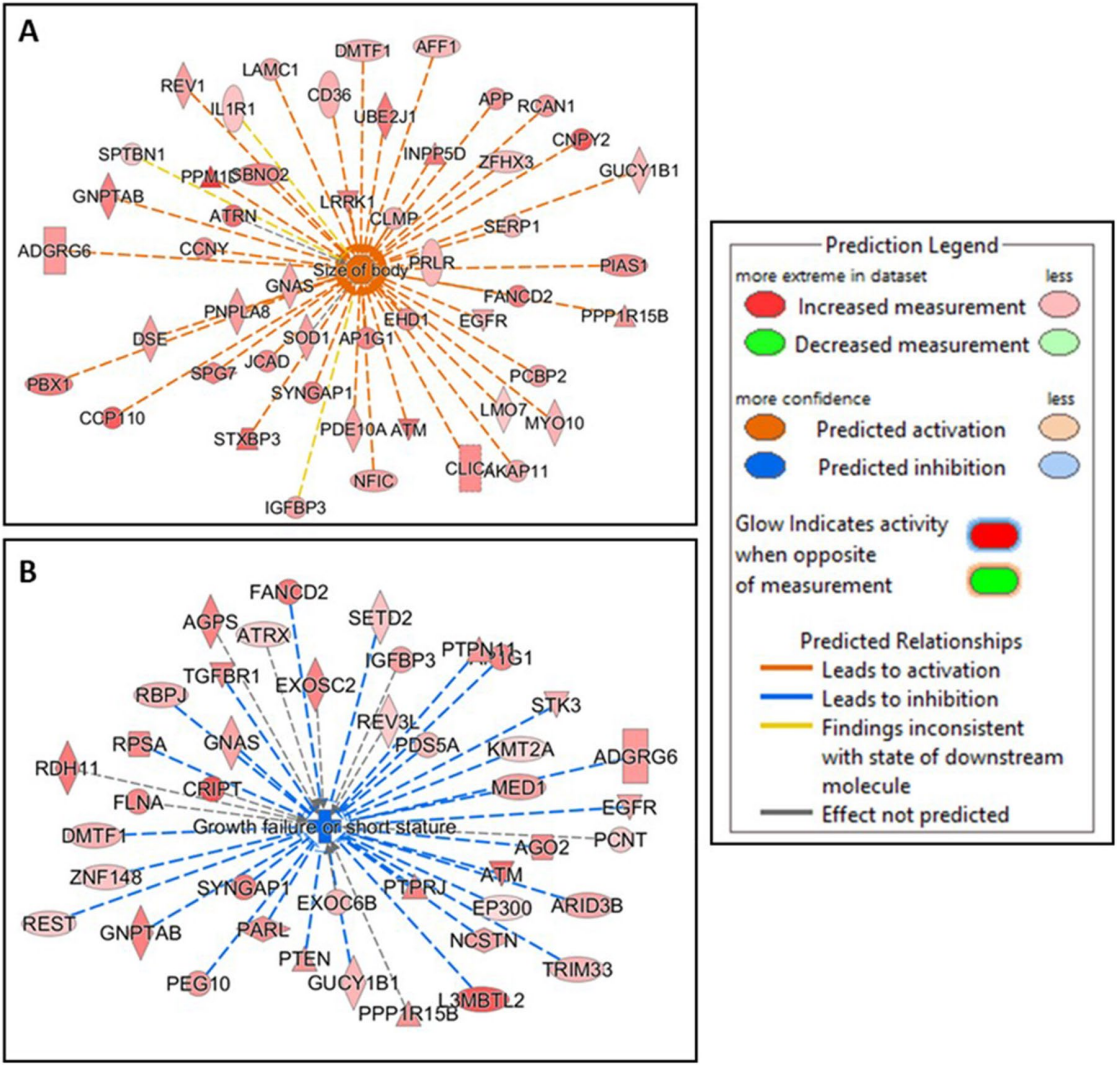
Diseases and functions predicted to be activated or inhibited in response to underweight status. In male placentas, underweight status was associated with several genes displaying measurement directions consistent with a significant (**A**) activation of size of body and (**B**) inhibition of growth failure or short stature.

Diseases and functions were predicted to be activated or inhibited by mRNAs associated with underweight status in male placentas. Notably, mRNAs with higher expression levels in relation to underweight status were predicted to increase activation of 51 diseases or functions and decrease activation of 54 diseases or functions (Table S5). Size of body (z-score 6.382, p-value = 3.29E−05) was among the top ten diseases or functions predicted to be increased, with 47 of 53 genes having measurement direction consistent with increase in size of body (Fig. 2A). Growth failure (z-score = − 6.092, Overlap p-value = 7.81E−05) and growth failure or short stature (z-score = − 6.077, Overlap p-value = 8.20E−06) were among the top ten diseases or functions predicted to be decreased, with 38 of 40 and 38 of 49 genes having measurement direction consistent with decreases in these functions, respectively (Fig. 2B).

### Pre-pregnancy underweight status was associated with placental miRNA expression levels in a sexually dimorphic manner

**Figure 3:**
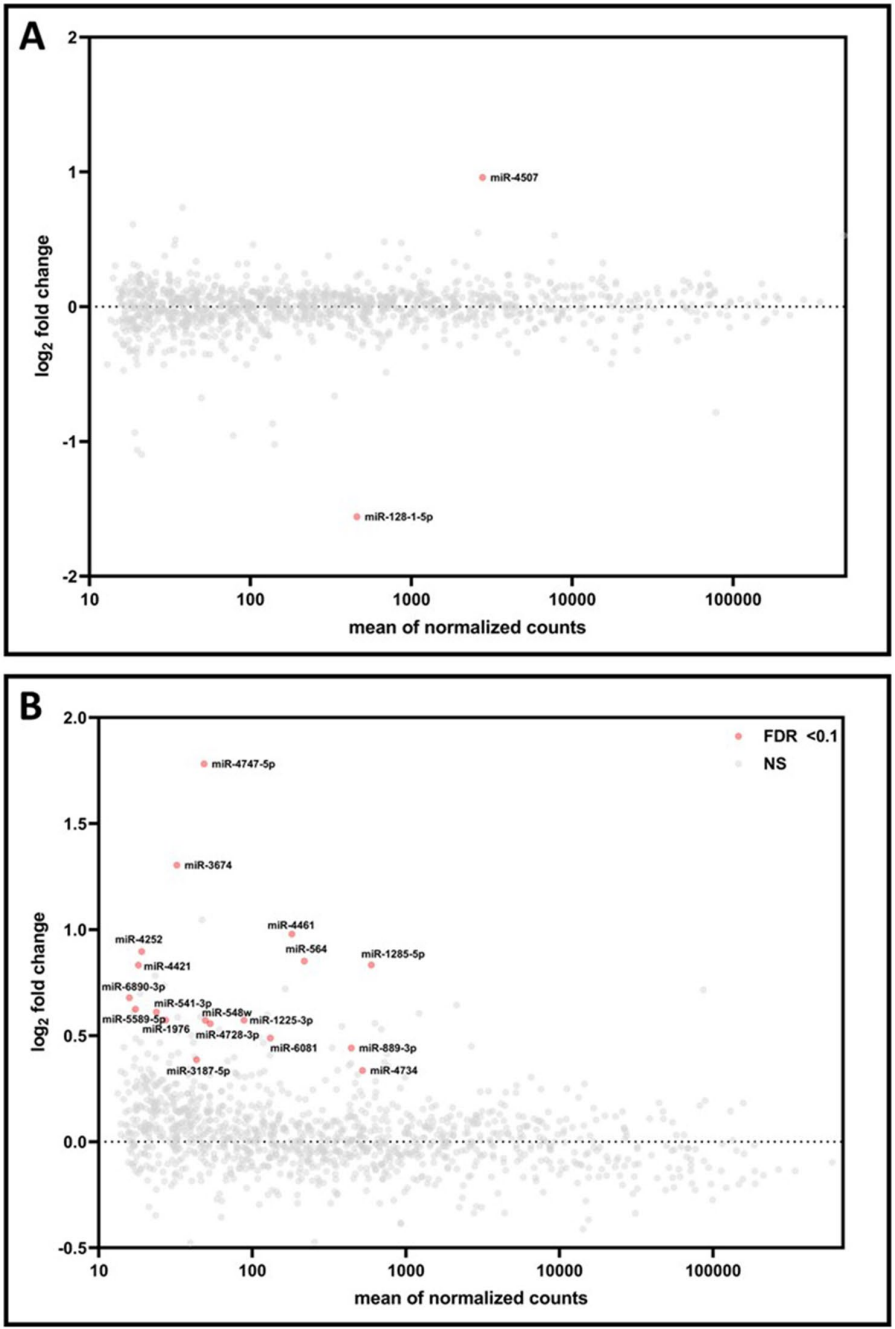
Log_2_-fold changes in miRNA expression associated with pre-pregnancy underweight status vs. mean of normalized counts. log_2_-fold changes represent the change in miRNA expression in (**A**) male and (**B**) female placentas. Data points colored in red indicate genes significantly (FDR p-value < 0.1) associated with underweight status. Genes not significantly associated with underweight status are displayed in grey.

Placental miRNA expression levels were also evaluated in relation to pre-pregnancy BMI. No miRNAs displayed expression levels that were significantly (FDR p-value < 0.10) associated with pre-pregnancy obesity. However, three miRNAs were differentially expressed in relation to overweight status overall (Table S6). Sex-stratified models revealed different sets of miRNAs differentially expressed in relation to overweight status in male (N = 5) and female (N = 2) placentas (Table S7). Notable miRNAs identified as associated with pre-pregnancy overweight status in male placentas included: *miR-146a-5p* (log_2_-FC = 2.31, FDR p-value = 0.08) and *miR-210-3p* (log_2_-FC = 0.63, FDR p-value = 0.02). A total of 14 miRNAs were identified with expression levels significantly associated with pre-pregnancy underweight status overall (Table S6). When stratifying by sex, different miRNAs displayed significant associations with underweight status in male (N = 2) (Fig. 3A, Table S7) and female (N = 18) placentas (Fig. 3B, Table S7).

**Figure 4:**
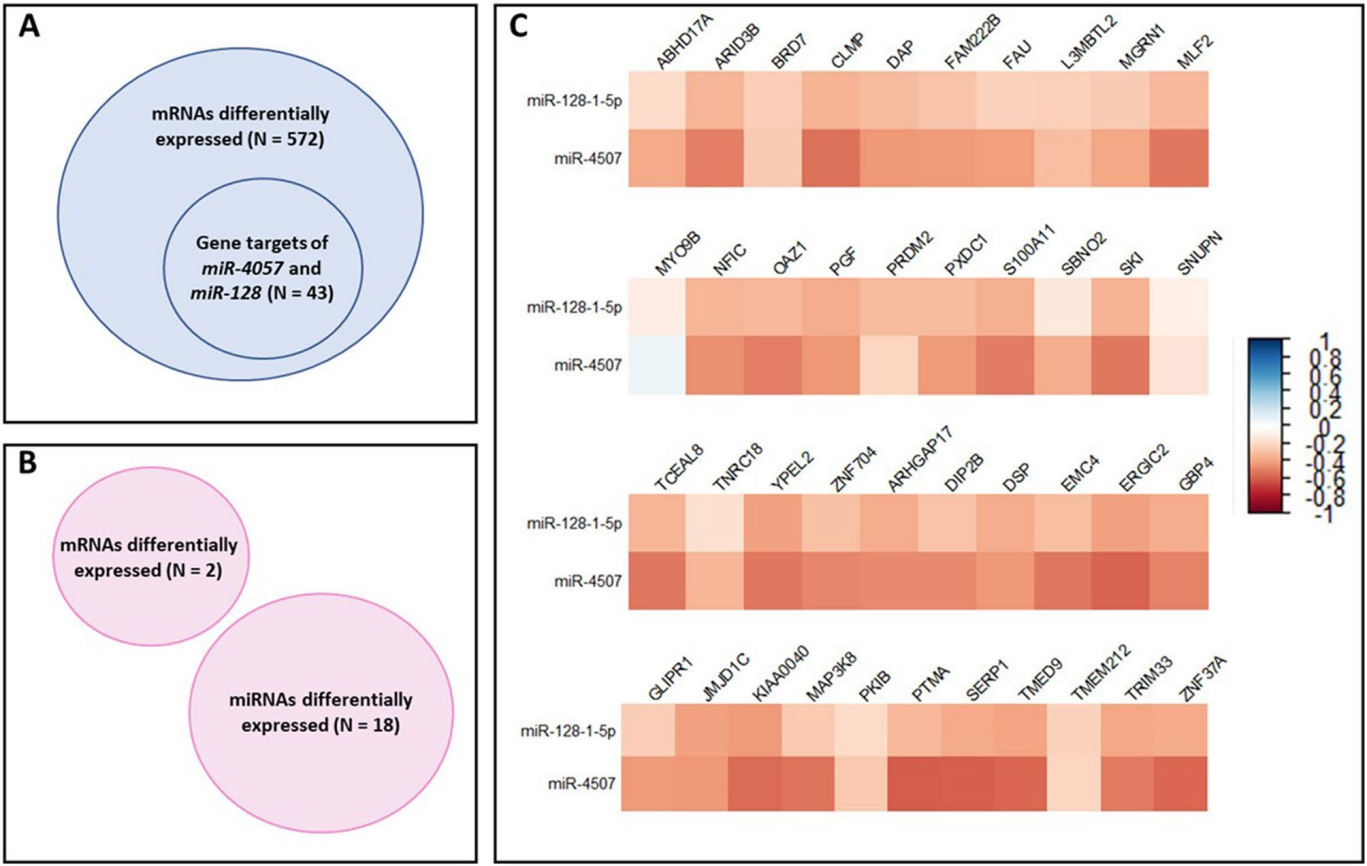
Potential epigenetic regulation of the association between underweight status and gene expression in male placentas. (**A**) 43/572 (8%) mRNAs associated with underweight status are also gene targets of two miRNAs (*miR-4057* and *miR-128-1-5p*) associated with underweight status in male placentas; (**B**) no mRNAs associated with underweight status are also gene targets of miRNAs associated with underweight status in female placentas; (**C**) higher miRNA expression was correlated (p < 0.05) with lower mRNA expression levels in 40/43 (90%) miRNA–mRNA expression pairings.

Interactions between miRNAs associated with underweight status and mRNAs associated with underweight status were predicted in male placentas in silico. Of the 572 mRNAs with expression levels associated with underweight status in male placentas, 43 (8%) were predicted to be gene targets of 2 miRNAs also associated with underweight status in male placentas, namely *miR-4057* and *miR-128* (Fig. 4, Table S8). Of the 43 miRNA–mRNA expression pairings, expression levels of 39 (90%) were inversely correlated (p < 0.05). More specifically, higher miRNA expression levels were associated with lower mRNA expression levels and lower miRNA expression levels were associated with higher mRNA expression levels, suggesting potential regulation of underweight-associated mRNAs by underweight-associated miRNAs.

## Discussion

Adverse birth outcomes associated with pre-pregnancy BMI vary according to BMI categories, with underweight women at increased risk of delivering infants preterm, having IUGR or low birth weight^1,2,4^. There is also evidence of these outcomes impacting males and females differentially, with IUGR more prevalent among females^20^. The placenta connects mother and fetus and is known to display sexually dimorphic patterns of gene expression, protein expression, and other physiological functions^8–11^. We hypothesized that pre-pregnancy BMI would also be associated with placental mRNA and miRNA expression in a sexually dimorphic manner. Genome-wide mRNA and miRNA expression levels were measured in placental tissues and evaluated in relation to pre-pregnancy BMI. In total, 719 genes were differentially expressed in relation to pre-pregnancy underweight status. In contrast, no genes were differentially expressed in relation to overweight or obese status. Sex-stratified models revealed that underweight status was associated with differential expression of 572 genes in placentas collected from male births, supporting our hypothesis of sexual dimorphism. Canonical pathways underlying nutrient transport, glucose metabolism, and angiogenesis were enriched for genes displaying higher expression levels in relation to underweight status in male placentas, and downstream effects included significant increases in body size and significant decreases in growth failure. With relevance to neonatal outcomes, these findings suggest that (1) the relationship between pre-pregnancy BMI and placental gene expression is sexually dimorphic and (2) the male placenta may adapt to underweight status or low nutrient environments by altering gene expression.

The relationship between pre-pregnancy BMI and placental mRNA expression is sexually dimorphic. Overall, 719 genes were differentially expressed in relation to pre-pregnancy underweight status. Evaluating male and female placentas separately revealed that 572 genes were differentially expressed in male placentas and 2 genes were differentially expressed in female placentas. Building upon existing literature highlighting the placenta as a sexually dimorphic organ^8–11^, these findings suggest placental responses to pre-pregnancy BMI also vary according to fetal sex. To our surprise, no mRNAs were differentially expressed in relation to pre-pregnancy overweight or obese status. In prior work investigating the molecular link between maternal BMI and fetal growth, Cox et al*.* did not identify a significant association between maternal pre-pregnancy BMI and placental transcripts (14,040 genes) in a cohort of 183 mother-infant pairs^21^. Differences in findings concerning underweight status between their study and ours might be explained by differences in (1) study populations (full- vs. preterm); (2) power to detect effects among underweight women; and/or (3) gene expression profiling techniques (microarray vs. RNA-seq).

Canonical pathways with established links to fetal growth are enriched for genes associated with underweight status in male placentas. Specifically, genes whose ontology predicts an effect on upregulation of Mammalian Target of Rapamycin (mTOR) signaling, Eukaryotic Initiation Factor 2 (EIF2) signaling, Insulin-like Growth Factor 1 (IGF-1) signaling, and Vascular Endothelial Growth Factor (VEGF) signaling were highlighted in male placentas. These pathways are critical to fetal growth and development as they contribute to the regulation of placental amino acid transport and resource allocation to fetal growth^22–24^. In the present study, we found that genes displaying higher expression in relation to pre-pregnancy underweight status in male placentas were enriched for mTOR and EIF2 signaling pathways. This is noteworthy because placental mTOR and EIF2 signaling pathways are linked to the nutritional, metabolic, and physiological state of the mother, and altered amino acid transporter activity within the placenta is causally linked to changes in fetal growth^25,26^. Since altered mTOR and EIF2 signaling was only observed in relation to underweight status in male placentas, factors which impair fetal growth or nutrient delivery to the fetus may be associated with adaptive transcriptional responses in placentas belonging to males but not females. This may explain, at least in part, the potential difference in IUGR between sexes^20^. Work by Roos et al*.* supports this hypothesis. In evaluating the expression of a protein indicative of mTOR signaling activity, they found that mTOR signaling was markedly lower in placentas obtained from pregnancies complicated by IUGR^25^.

IGF-1 and VEGF signaling were also among pathways up-regulated in male placentas. IGF-1 signaling is responsible for mediating the growth-promoting effects of the pituitary growth hormone and is important for fetal growth and development^27^. Altered IGF-1 signaling in the placenta has been associated with altered intrauterine growth of the fetus. Specifically, a study by Iniguez et al*.*, investigating the IGF-1 signal transduction pathway, identified higher IGF receptor protein content and increased responses to IGF-1 in placentas collected from infants who were small-for-gestational age, representing a potential compensatory mechanism in response to fetal growth restriction^28^. This further supports our hypothesis that factors which impair fetal growth or nutrient delivery to the fetus may be associated with adaptive transcriptional responses in placentas belonging to males. Pathways underlying angiogenesis were also upregulated in male placentas. These include the VEGF signaling and VEGF Family Ligand–Receptor Interactions, which promote pro-angiogenesis activity^29^. Barut et al. found increased expression of VEGF in placentas complicated by IUGR^30^. The observed, increased expression and upregulation of these proteins suggests that abnormal angiogenic activation may relate to the pathophysiology of IUGR^30^. Altered biological signaling in the placenta may contribute to the risk of IUGR, low birth weight, and other growth-related anomalies in babies born to underweight women. Thus, it is noteworthy that in male placentas, mRNAs associated with maternal underweight status had measurements consistent with significant increases in body size and decreases in growth failure. This may contribute to, at least in part, the potential difference in IUGR risk between male and female neonates.

In evaluating the potential for epigenetic regulation by miRNAs, we found that underweight status was associated with the expression of two miRNAs in male placentas, namely *miR-4057* and *miR-128-1-5p*. Using an *in-silico* approach, 8% of mRNAs associated with underweight status in males were also predicted to be gene targets of these two miRNAs, suggesting marginal epigenetic regulation. In female placentas, 18 miRNAs were differentially expressed in relation to underweight status. Building upon research by our group^12^, differences in miRNAs associated with underweight status according to fetal sex provide evidence for sexual dimorphism in placental miRNA expression. Previous studies report significant associations between placental miRNA expression and maternal BMI^13,19^. Using a candidate gene approach in 211 mother-infant pairs, Tsamou et al*.* also reported sexual dimorphism in the relationship between placental miRNA expression and maternal BMI^13^. In their study, they found that *miR-210* was associated with maternal BMI in female placentas, with significant modification by gestational weight gain^13^. We similarly identify an association between *miR-210* and pre-pregnancy overweight status that is sexually dimorphic. While no mRNAs were associated with high pre-pregnancy BMI, it is notable that we replicate an association between *miR-210* and overweight status because this miRNA is implicated in hypoxia and oxidative stress and related to key processes underlying obesity^31–36^. Findings from our study replicate published findings while highlighting novel associations between placental miRNA expression and low pre-pregnancy BMI.

This study is among the first to demonstrate that placental mRNA and miRNA expression profiles are associated with pre-pregnancy underweight status. Several factors should be considered when interpreting the data presented here. First, the ELGAN cohort comprises infants born extremely premature (less than 28 weeks of gestation), and findings from the present study may not be generalizable to full-term infants. The lower sample size is likely to influence the precision of association estimates in relation to underweight status. Still, ELGAN represents one of the largest placental repositories integrating multi-omics data and contains sufficient data to inform hypothesis testing. Future research should aim to replicate these findings in larger cohorts. Second, BMI may demonstrate poor sensitivity in detecting body fat percentage-defined obesity and is influenced by factors that may also influence gene expression and resulting birth outcomes^37^. Nevertheless, it is routinely used in epidemiologic and clinical settings, allowing for comparison across studies. Additionally, while inverse associations between the expression of underweight-associated miRNAs and their corresponding gene targets were observed, we are currently unable to assess the effects on protein expression given that these data are currently not available for ELGAN.

The current study provides novel information about the association between pre-pregnancy BMI, placental function, and fetal development. The data presented here suggest that pre-pregnancy underweight status is associated with mRNA expression levels in a sexually dimorphic manner. While potential epigenetic regulation by miRNAs was predicted to be modest, we did replicate an association between pre-pregnancy overweight status and *miR-210* that has previously been reported^13^*.* In male placentas, altered gene expression may confer protection against IUGR, as downstream effects analyses revealed several genes with measurement directions consistent with significant increases in size of body and decreases in growth failure. The data suggest that the relationship between low pre-pregnancy BMI and placental gene expression is sexually dimorphic, with gene expression changes related to nutrient transport, growth, and angiogenesis observed in male placentas only.

## Methods

### Subject recruitment and BMI evaluation

The ELGAN cohort was established to identify factors contributing to the risk of adverse neurocognitive outcomes among infants born extremely preterm (pregnancy ending with a live birth prior to completing 28 weeks of gestation). Study procedures were approved by the Institutional Review Board at each of the participating institutions, and methods of subject recruitment and sample collection have been described elsewhere^38–40^. In short, from 2002 to 2004, women giving birth at one of the participating sites prior to completing 28 weeks of gestation were asked to enroll in the ELGAN study. Informed consent was obtained from all mothers, and all methods were performed in accordance with the Declaration of Helsinki. For mothers younger than 18, informed consent was obtained from a parent and/or legal guardian. In total, 1506 infants and 1249 mothers enrolled in the ELGAN study, and a subsample of N = 426 provided sufficient placental tissue for multiple -omics analyses. Maternal demographic and clinical characteristics were measured at or prior to the time of delivery. Each mother self-reported her height and pre-pregnancy weight prior to or after delivery when she was interviewed, and these data were used to calculate pre-pregnancy BMI^40^.

### Placental tissue collection

Delivered placentas were placed in a sterile examination basin and transported to a sampling room, with 82% percent of samples obtained within 1 h of delivery^39^. Further details on histologic analyses have been provided elsewhere^41,42^. Briefly, the area sampled was at the midpoint of the longest distance between the cord insertion and the edge of the placental disk. Amniotic tissue was pulled away from the underlying chorion using a sterile technique in order to expose the chorion. A tissue sample was cut out of the base of the chorion, the fetal component of the placenta, after applying traction to the chorion and the underlying trophoblastic tissue. Tissue samples were subsequently placed into a sterile 2 mL cryo-vial that was immediately submerged in liquid nitrogen. Samples were shipped to the University of North Carolina at Chapel Hill and stored at − 80 °C until further processing^39^. For the present study, a total of 390 placentas were available for subsequent RNA analyses based on RNA quality and tissue abundance.

### Placental RNA extraction and sequencing analyses

In processing placental samples, cryotubes were first placed on dry ice. A subsection of placental tissue (0.025 g) was cut from each frozen tissue sample using a sterile dermal curette and washed in 1 × PBS (Fisher Scientific, Waltham, MA) to remove any residual blood. To preserve sample integrity, washed samples were immediately snap frozen in homogenization tubes and placed on dry ice. Tissue segments were homogenized using a sterile stainless-steel bead (Qiagen, Germantown, MD) in RLT + lysis buffer (Qiagen) with the TissueLyserII instrument (Qiagen). Samples were spun to collect the bead and any cellular debris, with homogenate samples stored at − 80 °C until nucleic acid extraction. RNA molecules 18 nucleotides and greater were extracted using the AllPrep DNA/RNA/miRNA Universal kit (Qiagen). RNA quantity was measured using the NanoDrop™ 1000 Spectrophotometer (Thermo Scientific, Waltham, MA) and tested for quality based on RNA integrity scores produced by the QIAxcel system (Qiagen).

Using isolated RNA samples, genome-wide miRNA expression profiles were measured using the HTG EdgeSeq miRNA Whole Transcriptome Assay (HTG Molecular Diagnostics, Tucson, AZ). This assay uses next-generation sequencing technologies to analyze expression levels of N = 2083 human miRNA transcripts. The counts of sequencing reads per miRNA were aligned to miRBase v20 and organized using Parser (HTG Molecular Diagnostics). The isolated RNA samples were also used to measure genome-wide mRNA expression profiles using the QuantSeq 3′ mRNA-Seq Library Prep Kit (Illumina). Libraries were pooled and sequenced (single-end 50 bp) on one lane of the Illumina Hiseq 2500 and the counts of sequencing reads per mRNA were aligned to the GENCODE database v3^43^ and organized using Salmon^44^. This process yielded measures of 37,268 unique human RNA transcripts, including protein-coding and non-coding RNAs. The resulting summarized count data were then used in data processing and statistical analyses. The reliability of RNASeq results was confirmed through a series of additional quality control steps including (1) filtering lowly expressed mRNAs/miRNAs; (2) the inclusion of surrogate variables within statistical models to account for additional sources of heterogeneity across samples; (3) the confirmation that published mRNA and miRNA placental-specific clusters were captured in the current dataset, as described in detail and published in our previous study using the same data^12^.

### Statistical analyses of differential miRNA and mRNA expression

In total, 360 placentas were included in the mRNA and miRNA analyses. Subjects were removed from the mRNA and/or miRNA analyses if they met the following exclusion criteria: (i) low expression values (i.e., non-detection across all genes) (N = 2 for the mRNA analysis); (ii) identified as sample outliers through principal component analyses (PCA) (N = 2 for the mRNA analysis, N = 2 for the miRNA analysis); (iii) demographic data were missing for the included covariates (N = 26). miRNA and mRNA sequencing data were processed separately using packages within R (v3.6.2). Count data were first filtered to exclude universally lowly expressed transcripts, requiring that > 25% of the samples be expressed at signals above the overall median signal intensity. This resulted in the final inclusion of 10,412 mRNAs and 1131 miRNAs for the analyses. Potential sample outliers were identified via PCA and hierarchical clustering, using the prcomp and hclust functions. Count data were then normalized by median signal intensity using algorithms within DESeq2 (v1.24.0) to produce variance-stabilized expression counts^45^. Potential sources of sample heterogeneity, including batch effects, were accounted for using surrogate variable analysis (SVA) within the SVA R package (v3.32.1). This method uses default parameters to estimate control probes^45^. Three significant surrogate variables were calculated and included as covariates in the final model.

Demographic covariates with plausible associations with pre-pregnancy BMI and placental gene expression were included in the model: maternal race (White/non-White), smoking status (none/first- or secondhand), maternal age (continuous), and a summative proxy for socioeconomic status indicating (1) reliance on public assistance, (2) less than 12 years of education, and/or (3) publicly or uninsured status (0–3). These covariates were selected if they were significantly associated (*p* < 0.05) with the exposure and/or based on their a priori status as confounders using a directed acyclic graph (DAG) approach. Pre-pregnancy BMI was categorized into four bins, according to standards provided by the Centers for Disease Control and Prevention (CDC): underweight: < 18.5 kg/m^2^, normal: 18.5 to < 25.0 kg/m^2^, overweight: 25.0 to < 30.0 kg/m^2^, and obese: ≥ 30.0 kg/m^2^^46^. Normal weight was the referent to which each group (underweight, overweight, and obese) was compared.

Statistical methods incorporating negative binomial generalized linear models within the DESeq2 package were implemented to identify mRNAs and miRNAs with expression levels associated with pre-pregnancy BMI status. This method uses a Wald test and generates z-statistics based on the calculation of shrunken logarithmic fold changes in expression for each BMI category (e.g. overweight vs. normal) divided by their standard errors. Resulting z-statistics are compared against standard normal distribution curves, generating Wald test p-values. These p-values are then adjusted for multiple testing using the Benjamini and Hochberg (BH) procedure^47^. Separate models were run for mRNAs and miRNAs. Differentially expressed mRNAs and miRNAs were defined as those with false discovery rate (FDR) p-value < 0.10, based on a BH-adjusted p-value. Both mRNA- and miRNA-seq data were first analyzed for statistical relationships with pre-pregnancy BMI using placental data from all newborns. Next, a sex-stratified approach was used, analyzing placenta data derived from male newborns separate from female newborns to evaluate potential sexually dimorphic patterns.

### Identification of gene targets of miRNAs and correlation between miRNA–mRNA expression

An in silico approach was utilized to identify known miRNA–mRNA interactions among miRNAs and mRNAs that were both associated with pre-pregnancy BMI. The Ingenuity Knowledge Database (Ingenuity Systems^®^, Redwood City, CA), which couples experimentally observed miRNA–mRNA interactions curated from literature and computational predictions, was queried. First, known mRNA targets of the miRNAs identified with expression levels significantly associated with pre-pregnancy BMI were identified. To detail further, experimental observations were gathered from TarBase, which contains approximately 670,000 unique miRNA–mRNA interactions shown through published literature^48^. Computationally predicted interactions were derived using algorithms generated through TargetScan Human v7.2, which identifies miRNA–mRNA interactions based on potential base pairing homologies between the 3′ untranslated mRNA regions and miRNA seed sequences^49^. Second, resulting potential miRNA–mRNA interactions were filtered to include those that have been experimentally observed of those with high predicted confidence, measured as cumulative weighted context scores ≤ − 0.4. These scores represent an aggregation of factors influencing the likelihood of miRNA–mRNA interactions, including binding site type and location, local adenine and uracil content, target site abundance, seed-pairing stability, and supplementary pairing^50^. Third, predicted gene targets were filtered to include only those that were observed to be significantly associated with pre-pregnancy BMI. These steps resulted in a list of miRNA–mRNA predicted interactions. Thus, a miRNA–mRNA predicted interaction is defined hereafter as a miRNA and a mRNA in which (1) the mRNA is a predicted gene target of the miRNA and (2) both miRNA and mRNA were found to be significantly associated with BMI. Correlations between variance-stabilized miRNA and mRNA expression counts were quantified using Pearson correlations.

### Pathway enrichment analyses

Canonical pathway analyses were performed on mRNAs associated with underweight status among males only given that few (N = 2) genes were differentially expressed in female placentas. Using the Ingenuity Knowledge Database (Ingenuity Systems^®^, Redwood City, CA), we sought to identify the systems-level response to pre-pregnancy BMI status and uncover biological functions and diseases linked to mRNAs dysregulated in relation to underweight status. Over-represented canonical pathways were defined as those containing more pre-pregnancy BMI-associated mRNAs than expected by random chance, as based on a BH-corrected p-value calculated from a right-tailed Fisher’s Exact Text^47,50^. Expression analyses first considered molecules and/or relationships where “(confidence = Experimentally Observed)”. Next, molecules and/or relationships where “(confidence = Experimentally Observed) AND (tissues = Placenta)” were considered. Pathways with enrichment BH-corrected p-values < 0.05 were considered significant.

### Ethics approval and consent to participate

Each participant provided consent either upon hospital admission prior to or shortly after delivery. All procedures were approved by the Institutional Review Board at each participating study site, specifically: North Carolina Children's Hospital (Lead Site), Baystate Children's Hospital, Brenner Children's Hospital, Boston Children’s Hospital, Floating Hospital For Children (Tufts), Helen Devos Children's Hospital, Michigan State University and Sparrow Hospital, UMASS Memorial Hospital, University Of Chicago Medical Center, East Carolina University-Brody School Of Medicine, William Beaumont Hospital, and Yale-New Haven Children's Hospital.

## Supplementary Information

Supplementary Tables.

## Data Availability

The datasets used and/or analysed during the current study are available at https://www.ncbi.nlm.nih.gov/geo/query/acc.cgi?acc=GSE167885.
